# Commentary: Leaflet fluttering of bioprosthetic valve—Does it matter?

**DOI:** 10.1016/j.xjon.2020.10.009

**Published:** 2020-11-16

**Authors:** Dominik Obrist, Thierry P. Carrel

**Affiliations:** a ARTORG Center for Biomedical Engineering Research, University of Bern, Bern, Switzerland; b Department of Cardiovascular Surgery, Inselspital, Bern University Hospital, University of Bern, Bern, Switzerland

**Figure:**
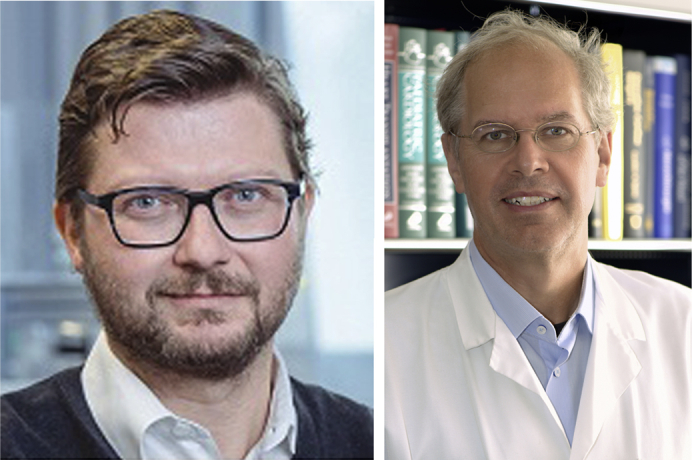
Dominik Obrist, PhD, and Thierry P. Carrel, MD

**Central Message**

Although bioprosthetic leaflet fluttering is scientifically intriguing, so far there is no proof that this phenomenon is of any clinical relevance (eg, tissue valve degeneration, blood cell damage). Nevertheless, an improved understanding of leaflet fluttering may lead to improved valve designs.

See Article page 60.

Systolic fluttering of bioprosthetic valve leaflets in aortic position has been reported in numerous studies, with typical fluttering frequencies in the range of 20 to 60 Hz and amplitudes of up to several millimeters. But although bioprosthetic leaflet fluttering is scientifically intriguing, we may ask the legitimate question of whether this phenomenon is of any clinical relevance or whether it is just an epiphenomenon of systolic blood flow. This question has remained unanswered so far.

Thus, it is remarkable that 3 studies have been published over the past few weeks that may provide some answers. In addition to the study reported by Lee and colleagues^1^ in this issue, which analyzes the relationships among leaflet fluttering frequency, valve size, and leaflet tissue thickness, 2 independent studies that also used sophisticated computational models have provided concordant findings.^2^^,^^3^ The results of all 3 studies emphasize that leaflet fluttering is more relevant to bioprosthetic valve performance and durability than commonly believed.

Limited valve durability and structural deterioration have been related in part to mechanical fatigue of the leaflet tissue, which undergoes hundreds of millions of cycles during the valve's lifetime. Whereas the maximum mechanical stress on the leaflet tissue due to fluttering is lower than the stress on the closed valve, the number of load cycles during fluttering is significantly higher than the number of diastolic load cycles, because fluttering leaflets can experience more than 10 oscillations in every heartbeat. Therefore, reduced durability due to material fatigue may be directly related to increased fluttering frequency and fluttering amplitude. Lee and colleagues found that fluttering frequency decreases with valve size and increases with leaflet thickness, as shown in their Figure 4, whereas Johnson and colleagues^2^ reported that valves with thinner leaflet tissue tend to have higher fluttering amplitudes. These results suggest that the increasingly thinner and more flexible tissues used in transcatheter valves^4^ may be more prone to early wear and structural deterioration.

In addition to mechanical fatigue, bioprosthetic leaflet fluttering also may be related to damage to blood cells from turbulent blood flow. Although thrombogenicity is a problem seen mainly in mechanical valves, recent studies provide increasing evidence that the turbulent flow behind bioprosthetic valves also may activate thrombocytes. Becsek and colleagues^3^ directly correlated leaflet fluttering with the onset and intensity of turbulent flow, which is supported by the observations of Johnson and colleagues,^2^ who found increasingly disturbed flow for thinner leaflets with higher fluttering amplitudes. Becsek and colleagues further showed that turbulent flow may lead not only to shear-induced thrombocyte activation, but also to unphysiological wall shear stresses in the ascending aorta and to increased transvalvular pressure gradients. Therefore, leaflet fluttering may contribute to multiple adverse events that can affect the long-term outcome of aortic valve replacement.

Taken together, the results of these 3 studies indicate that bioprosthetic leaflet fluttering is a mechanical phenomenon that has clinical relevance, being related to valve durability, thrombogenicity, transvalvular pressure gradients, and even adverse aortic events. Future research should focus on the underlying physical mechanisms of leaflet fluttering to provide a better understanding of the main factors determining the intensity of fluttering. This improved insight can provide guidance for improved valve designs aimed at reducing leaflet fluttering.
